# Genome-wide gene expression profiling of testicular carcinoma *in situ* progression into overt tumours

**DOI:** 10.1038/sj.bjc.6602560

**Published:** 2005-04-26

**Authors:** K Almstrup, C E Hoei-Hansen, J E Nielsen, U Wirkner, W Ansorge, N E Skakkebæk, E Rajpert-De Meyts, H Leffers

**Affiliations:** 1 University Department of Growth and Reproduction, Rigshospitalet, Section GR-5064, Blegdamsvej 9, Copenhagen DK-2100, Denmark; 2 EMBL Heidelberg, Biochemical Instrumentation, Meyerhofstrasse 1, Heidelberg D-69117, Germany

**Keywords:** gene expression, testicular cancer, carcinoma *in situ*, seminoma, nonseminoma, *DNMT3L*

## Abstract

The carcinoma *in situ* (CIS) cell is the common precursor of nearly all testicular germ cell tumours (TGCT). In a previous study, we examined the gene expression profile of CIS cells and found many features common to embryonic stem cells indicating that initiation of neoplastic transformation into CIS occurs early during foetal life. Progression into an overt tumour, however, typically first happens after puberty, where CIS cells transform into either a seminoma (SEM) or a nonseminoma (N-SEM). Here, we have compared the genome-wide gene expression of CIS cells to that of testicular SEM and a sample containing a mixture of N-SEM components, and analyse the data together with the previously published data on CIS. Genes showing expression in the SEM or N-SEM were selected, in order to identify gene expression markers associated with the progression of CIS cells. The identified markers were verified by reverse transcriptase–polymerase chain reaction and *in situ* hybridisation in a range of different TGCT samples. Verification showed some interpatient variation, but combined analysis of a range of the identified markers may discriminate TGCT samples as SEMs or N-SEMs. Of particular interest, we found that both *DNMT3B* (DNA (cytosine-5-)-methyltransferase 3 beta) and *DNMT3L* (DNA (cytosine-5-)-methyltransferase 3 like) were overexpressed in the N-SEMs, indicating the epigenetic differences between N-SEMs and classical SEM.

The incidence of testicular germ cell tumours (TGCTs) has increased markedly during the past decades, and although accounting for only 1–2% of all cancers in men, TGCTs are now the most common malignancy in the age group of 15–35 years old men (Adami *et al*, 1994; Huyghe *et al*, 2003). Owing to a refined treatment, in particular the introduction of cisplatin in combination chemotherapy, mortality rates have declined rapidly (Einhorn, 1981; Schmoll *et al*, 2004). Despite the high survival rate, still 4–8% of relatively young patients die of the disease, especially those with disseminated nonseminomas (N-SEM).

Testicular germ cell tumours comprise a heterogeneous group of tumours, which are divided into two main histological subtypes, classical seminoma (SEM) and N-SEMs. The N-SEM can be further subdivided into choriocarcinoma, yolk sac tumour, embryonal carcinoma and teratomas, and several of these components are frequently present within one tumour. Despite the heterogeneity, virtually all TGCTs originate from a common precursor, the carcinoma *in situ* (CIS) cell (Skakkebaek, 1972), which is believed to arise by transformation of a gonocyte (Skakkebaek *et al*, 1987), and shows a striking similarity to embryonic stem cells at the gene expression level (Almstrup *et al*, 2004). In addition, epidemiological evidence (Moller, 1993; Bergstrom *et al*, 1996) and comparative immunohistochemical studies of expressed proteins/antigens (Rajpert-De Meyts *et al*, 2003) indicate that CIS is an inborn lesion, probably arising in early foetal life and progressing to an overt TGCT after puberty. The molecular mechanisms of the initial malignant transformation into a CIS cell, and the subsequent progression into overt tumours remains largely unknown.

Gene expression studies provide a useful tool to identify mechanistic pathways. A range of studies has recently reported gene expression patterns in overt TGCT. Several studies have focused on genes on chromosomes 17 and 12, because of frequent rearrangements of these chromosomes (Skotheim *et al*, 2002; Rodriguez *et al*, 2003; Skotheim and Lothe, 2003). One study reported gene expression patterns of testicular SEM compared to normal testicular parenchyma (Okada *et al*, 2003), and another study have reported gene expression in SEM- and embryonic carcinoma (EC)-derived cell lines (Sperger *et al*, 2003). Our investigations have so far focussed on the preinvasive CIS stage of TGCTs, and we have recently reported the genome-wide gene expression profile of CIS (Almstrup *et al*, 2004; Hoei-Hansen *et al*, 2004a).

In order to investigate the transition from the preinvasive CIS stage into overt tumours, we decided to analyse gene expression in the two main types of overt TGCTs derived from CIS cells, SEM and N-SEMs. We have used a microarray covering nearly the entire human transcriptome to investigate differences in gene expression between testicular tissue with CIS, a classical SEM and a N-SEM containing a mixture of various tumour components (embryonal carcinoma, yolk sac tumour and choriocarcinoma). The generated expression profiles were analysed together with published data on CIS using the same microarray (Almstrup *et al*, 2004). Identified differentially expressed genes were further verified by reverse transcriptase–polymerase chain reaction (RT–PCR) in a panel of TGCT and the cellularity of the expression assessed by *in situ* hybridisation (ISH). RT–PCR showed some interindividual variation of expression, but the number of markers identified allowed us to discriminate between SEM and N-SEM samples.

## MATERIALS AND METHODS

### Testicular tissues

The testicular tissue samples were obtained directly after orchidectomy and macroscopic pathological evaluation. The Regional Committee for Medical Research Ethics in Denmark approved the use of the orchidectomy samples for the studies of novel genes expressed in germ cell cancers. Samples of homogeneous overt testicular tumours were excised and divided into several tissue fragments. Two to three fragments were snap-frozen and stored at −80°C for nucleic acid extraction, and several adjacent fragments were fixed overnight at 4°C in Stieve's fluid or paraformaldehyde (PFA), and then embedded in paraffin. Fixed sections were subsequently stained with haematoxylin and eosin (H&E) or by immunoperoxidase method for placental alkaline phosphatase (*ALPP* or *PLAP*) (Giwercman *et al*, 1991), in order to obtain the closest approximation of the histological content of the samples.

Besides the histological classification of the adjacent fragments, we also made imprints of the frozen fragments that specifically were used for RNA isolation. This was an attempt to circumvent the uncertainty about differences in cellularity between adjacent biopsies from the same patient. After staining with ALPP (PLAP) antibody, we could roughly confirm the histological observations seen in the adjacent fragments (data not shown). A histological description of all the used samples is presented in Table 1 with abbreviated names.

### Stimulation of differentiation in cell lines

Two established embryonal carcinoma-derived cell lines, NT2 and 2102Ep cells (Andrews, 1984), were grown in standard conditions (5% CO_2_, 37°C). For analysis of the effect of retinoic acid (RA) on gene expression, NT2 cells were grown in DMEM medium (10% FBS, 2 mM L-glutamine, 25 IU ml^−1^ penicillin, 25 *μ*g ml^−1^ streptomycin) and stimulated with 10 *μ*M RA (Sigma-Aldrich, St Louis, MO, USA) for 0–15 days to induce differentiation. 2102Ep cells were grown in DMEM medium with added 100 *μ*M *β*-mercaptoethanol, and stimulated with 10 *μ*M RA for 0–10 days.

### Microarray analysis

Isolation, labelling and hybridisation of testicular RNA was carried out essentially as described before (Almstrup *et al*, 2004). In brief, total RNA was purified using the NucleoSpin RNAII kit as described by the manufacturer (Macherey-Nagel, Düren, Germany) and analysed on a Bioanalyzer 2100 (Agilent Technologies, Palo Alto, CA, USA). Samples of sufficient RNA quality were linearly amplified using the RiboAmp RNA amplification kit (Arcturus GmbH, Germany) and subsequently labelled with Cy3 and Cy5 using the Atlas Glass Fluorescent labelling kit (BD Biosciences Clonetech, CA, USA). Labelled probes were cohybridised to a 52 000 element cDNA microarray representing the entire Unigene database. Production of the microarray is described elsewhere (http://embl-h3r.embl.de/). Slides were scanned on a GenePix 4000B scanner (Axon Instruments, Union City, CA, USA) and the generated images analysed in ChipSkipper (http://chipskipper.embl.de/) using histogram segmentation. Unreliable flagged spots and controls were taken out and quantified spots were normalised using a framed median ratio centring (frame=200 genes). Each measurement relies on the average of dye-swap experiments and spots that showed a big standard deviation was sorted out. The data were analysed in the program Genesis (Sturn *et al*, 2002) where clustering was carried out.

### RT–PCR and ISH

In order to verify the results obtained from the microarray analysis, expression of a selected set of genes, which in the microarray analysis showed specific expression in either SEM or N-SEM, was verified by RT–PCR and ISH. In addition, expression of genes that could be associated with cell differentiation was analysed by RT–PCR in the NT2 and 2102Ep cell lines with and without RA treatment.

RT–PCR analysis was carried out essentially as described before (Almstrup *et al*, 2004; Hoei-Hansen *et al*, 2004a). In brief, total RNA was purified as described above, DNAse digested, and cDNA was synthesised using a dT20 primer. Specific primers were designed for each mRNA preferentially spanning intron–exon boundaries to avoid amplification of genomic DNA. However, in some cases additional primer pairs where both primers were localised in the 3′-exon were designed. PCR was performed in 30 *μ*l of (final concentrations): 12 mM Tris-HCl, pH 8.3; 50 mM KCl; 1.9 mM MgCl_2_; 0.1% Triton X-100; 0.005% gelatin; 250 *μ*M dNTP; and 30 pmol of each primer. H_2_O was used as a negative control, and *β*-actin (*ACTB*) and *β*2-microglobulin (*B2M)* were used as control of the PCR protocol. Cycle conditions: one cycle of 2 min at 95°C; 30–40 cycles (depending on the intensity of bands) of: 30 s at 95°C, 1 min at 62°C, 1 min at 72°C and finally one cycle of 5 min at 72°C. PCR products were run on 2% agarose gels and visualised by ethidium bromide staining. In a few of the RT–PCR analyses of less abundant transcripts, no bands were detectable after the first round of PCR and nested primers were designed. A measure of 1 *μ*l from the first PCR reaction was transferred to a new reaction containing the nested primers and analysed as above with 10–20 additional cycles.

Nested primers with additional T3 or T7 extension were used to generate PCR fragments, which subsequently were used in *in vitro* transcription reactions to generate sense and antisense RNA fragments used for the ISH as described before (Nielsen *et al*, 2003; Almstrup *et al*, 2004; Hoei-Hansen *et al*, 2004a).

## RESULTS

### Comparison of CIS with SEM and N-SEM

We isolated testicular RNA from two patients characterised as having a mixture of N-SEMs and a classical SEM, respectively, and compared gene expression in these samples to each other and to a sample from a patient classified as having CIS cells in all of the seminiferous tubules (100% CIS) (see Table 1). A triangular design was used and facilitated comparison between N-SEM *vs* 100% CIS, SEM *vs* 100% CIS and N-SEM *vs* SEM. The 100% CIS sample had previously been compared to a patient sample with complete and normal spermatogenesis (Almstrup *et al*, 2004), and the CIS data from this study were included in the subsequent data analysis. Data were filtered to highlight genes up- or downregulated in either the N-SEM or SEM as illustrated in Table 2. There was a noticeable difference in the cutoff values used in the filtering process to generate gene lists specific for either SEM or N-SEM. A lower cutoff value (three-fold in the SEM group compared to five-fold in the N-SEM group) was used to display a reasonable number of SEM-specific genes. This lower cutoff value probably reflects the fact that CIS cells are more similar to SEMs than N-SEMs (Nielsen *et al*, 1974; Albrechtsen *et al*, 1982; Gondos, 1993). In addition to the identification of genes specifically upregulated in SEM and N-SEM, we in a similar manner searched for genes specifically downregulated in each of the two tumour types, aiming at identifying genes specifically downregulated during the invasive transformation of CIS into a SEM or N-SEM, respectively.

**Figure 1:**
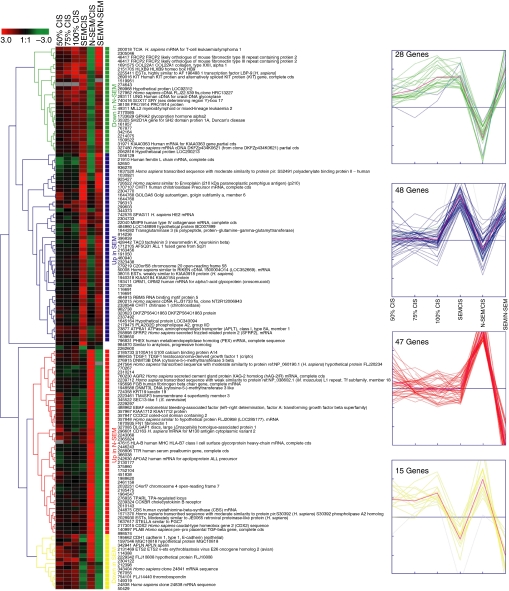
Hierarchical and *K*-means clustering of genes expressed specifically in testicular SEM or N-SEM. Results from the microarray analysis were filtered to show genes whose expression would discriminate between the two overt tumour types. This list was then subjected to hierarchical and *K*-means clustering (*K* was set to 4 according to the number of groups filtered for) using Euclidian distance measures. The clustering was carried out using the Genesis software (Sturn *et al*, 2002). Results on the CIS samples are from Almstrup *et al* (2004).

The expression data of the selected genes were subjected to hierarchical clustering with average linkage as shown in Figure 1. In addition, a *K*-means clustering was made with *K* set to equal the four filter groups. The *K*-means clustering nearly completely resolved into the initial four filter groups, indicating a good separation between the groups (Figure 1).

### Verification by RT–PCR and ISH

**Figure 2:**
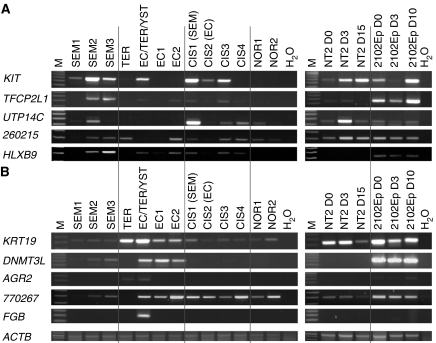
Verification of the microarray data by RT–PCR. RT–PCR primers for selected genes were used on a panel of different testicular tissue samples and cell lines. Gene-specific primers were designed preferentially to span intron–exon boundaries. The genes were divided according to the microarray analysis into: (**A**) Genes preferentially overexpressed in SEMs and (**B**) genes preferentially overexpressed in N-SEM. At the lower part of the figure, expression of *ACTB* is shown as a control of the RT–PCR protocol. Sample abbreviations can be found in Table 1, except for: M=100 bp marker, H_2_O=control, NT2=NT2 cell line days 0–15 after RA treatment, 2102Ep=2102Ep cell line days 0–10 after RA treatment.

In order to verify the results obtained from microarray analysis, the expression of several differentially regulated genes was further analysed by RT–PCR (Figure 2). This was performed on a spectrum of different TGCTs to allow not only verification of the expression in the samples used in the microarray analysis but also in other specimens from patients with similar diagnosis (Figure 2; Table 1). The results showed some variation, even between samples with the same histological diagnosis. Differences in the cellularity of the fragments used for RNA isolation may play a role in this, but could not alone explain the observed variation, suggesting that gene expression was variable also within samples from similar tumour types. Bioinformatic searches in various databases (i.e. http://www.ensembl.org/) revealed that for many loci additional transcripts were described, including transcripts from both strands (e.g. *HLXB9*, *TAC3*, *SEC13*, *CHIT1* and *COL22A*), which could influence the results. For most genes, we amplified PCR fragments across splice sites in order to avoid amplification of genomic DNA, but for some genes, we also applied primer pairs positioned internally within the 3′-exon (which typically corresponded to the PCR fragment on the array). For many of the genes that were assayed with both intron–exon spanning and 3′-exon primer pairs, different results were obtained (data not shown) substantiating the presence of multiple transcripts. Interestingly, we found a high expression of fibrinogen beta B (*FGB*) gene in a sample of N-SEM with various components, but not in pure EC or teratomas.

**Figure 3:**
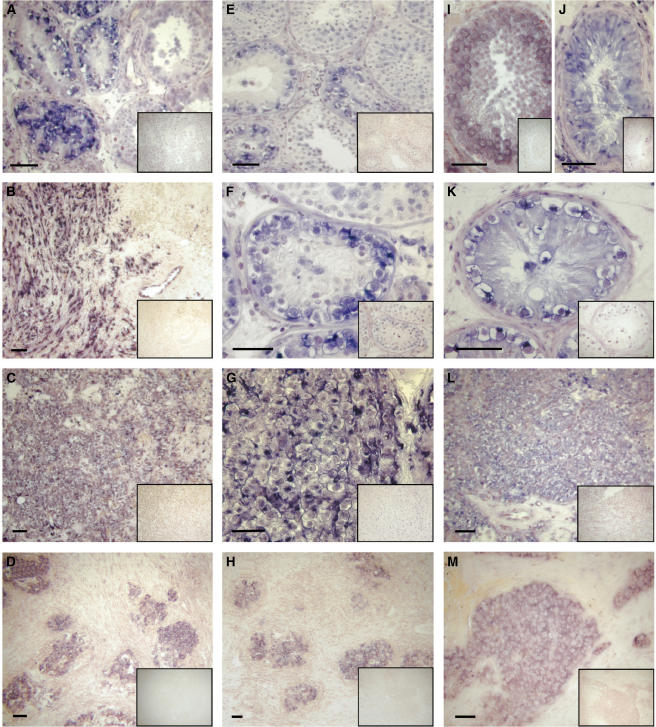
Identification of the expressing cell types by ISH. *In situ* hybridisation was performed with antisense and control sense (inserted images) RNA probes. Expression is shown for three genes: *KIT* (**A**–**D**), *HLXB9* (**E**–**H**), and the IMAGE clone number 260215 (**I**–**M**). *KIT* was highly expressed in CIS cells (**A**) and in SEM (**C**), and present in components of teratoma, for example, keratinised intestine-like epithelium as shown here (**B**) and embryonal carcinoma (**D**). *HLXB9* expression was high in CIS cells (**E**+**F**) and SEM (**G**); *HLXB9* was present in embryonal carcinoma tumour components (**H**) and it was detected in spermatocytes, spermatids and Leydig cells in normal testicular tissue (**E**). IMAGE clone 260215 was present in spermatocytes, spermatids and Sertoli cells in normal testicular tissue (**I**); the expression in Sertoli cells was confirmed in a tubule with Sertoli cell-only pattern (**J**). Additionally, the transcript was highly expressed in CIS cells (**K**) and present in SEM (**L**) and components of embryonal carcinoma (**M**). Scale bar represents 50 *μ*m.

The cellularity of the expression was assayed by ISH (Figure 3). In general, expression was localised to the neoplastic tissue. However, in accordance with the presence of multiple transcripts, we some times observed hybridisation signals in tissues that by microarray and RT–PCR analysis appeared to have low or even nondetectable expression of the gene (i.e. *KIT*; Figures 2A and 3A–D). In addition, we occasionally observed hybridisation signals from both the antisense and sense probes or only from the sense probe. Together, this strongly suggests the presence of transcripts derived from both strands and could indicate that results from RT–PCR, ISH and microarray analyses were not always derived from the same transcripts (see Discussion).

### Expression in cell lines

In the RT–PCR analysis, we used two embryonal carcinoma cell lines to investigate changes of expression related to tumour differentiation (NT2 cells that differentiate after stimulation with RA, and 2102Ep cells that do not differentiate upon RA addition). For a few genes (i.e. *KIT* and IMAGE clone 770267), the expression was affected by differentiation (Figure 2A).

## DISCUSSION

This is the first study aimed at investigating genome-wide gene expression changes during progression from preinvasive CIS to overt TGCTs. The results showed some interpatient variation, and a relatively small number of genes were differentially expressed between SEM and N-SEM was identified. This was probably caused by the experimental design of the microarray study as it only included one sample from each tumour type. The subsequent RT–PCR and ISH analysis on a range of samples showed some interpatient variation (Figures 2 and 3), especially for the nonseminomatous tumours. Similar variation in gene expression between testicular SEMs has been reported earlier (Okada *et al*, 2003). In the study by Okada *et al* (2003), the criterion for a SEM marker was an upregulation in just seven out of 13 microdissected SEM samples investigated. Their subsequent RT–PCR verification showed a variation similar to the one we have observed here, both in SEMs and N-SEMs, suggesting that the phenotypic plasticity is an intrinsic feature of TGCTs. Many of the genes that showed interpatient variation are listed in databases (i.e. http://www.ensembl.org/), with ESTs on both strands illustrating that there are expressed transcripts that do not correspond to the known genes in these regions. This is consistent with recent reports on widespread expression of transcripts from both strands in large parts of the human genome (Kapranov *et al*, 2002; Cawley *et al*, 2004), and we speculate that this feature may contribute to the heterogeneity of overt tumours – especially N-SEMs. Even protein expression has earlier been reported to be markedly heterogeneous in TGCTs, including CIS (Rajpert-De Meyts *et al*, 1996; Lifschitz-Mercer *et al*, 2002; Hoei-Hansen *et al*, 2004b; Kato *et al*, 2004). It is, however, evident from the RT–PCR results shown in Figure 2 that by combining results from a range of different genes, a TGCT sample could be classified as a SEM or N-SEM.

The CIS samples included in the microarray data analysis originated from tissue next to different overt tumours (Almstrup *et al*, 2004). It has been suggested that CIS cells next to SEMs and N-SEMs might be different (Oosterhuis *et al*, 1993; Rajpert-De Meyts *et al*, 1996). The reported expression in CIS is not specific for CIS cells next to a specific tumour type but represents an ‘average’ if differences exist between the analysed CIS samples. Some of the genes identified here have earlier been reported as differentially expressed (mainly at the protein level) along with CIS progression into overt tumours. An example of a gene with differential expression in various TGCTs is the v-kit Hardy–Zuckerman 4 feline sarcoma viral oncogene homologue, better known as KIT. The KIT protein is in most cases found expressed in CIS and SEM, but not in N-SEMs, with an exception of undifferentiated somatic elements that sometimes can be present in teratomas (Strohmeyer *et al*, 1991; Rajpert-De Meyts and Skakkebaek, 1994). KIT has, however, also been reported to be expressed in one-third of N-SEMs (Izquierdo *et al*, 1995), reflecting the heterogeneity of these tumours. In our microarray analysis, we found *KIT* in the ‘Down in N-SEM’ group (Figure 1), and the RT–PCR analysis in tumour samples confirmed this expression pattern (Figure 2A). However, a band was observed in the EC/TER/YST sample (Figure 2A), but not in the pure EC samples whereto ISH on the contrary revealed a faint hybridisation (Figure 3D; the samples used for RT–PCR and ISH were not from the same patient). On the other hand, a strong expression of *KIT* was observed in a partially keratinised epithelial component of teratoma (Figure 3B). There might be differences between expression of the *KIT* transcript and the reported protein expression, but the results most probably reflect the heterogeneity of N-SEMs.

In the ‘Up in N-SEM’ group (Figure 1), we found a range of genes known to be highly expressed in embryonic stem cells and EC cell lines and important for proper early development. These genes include *STELLA*, *CDX2*, *TDGF1* (Cripto) and *EBAF*, and emphasise the embryonic features of the N-SEMs. However, none were highly expressed in CIS cells (Almstrup *et al*, 2004), and thus apparently discriminate the embryonic features of CIS cells and N-SEMs. In addition, we identified a high expression of *FGB* gene, which may be a new marker for yolk sac tumour component of mixed TGCTs (Figures 1 and 2).

Gene expression in EC and SEM cell lines compared to embryonic stem cell lines has been described by Sperger *et al* (2003) and their results show some overlap with the genes we have identified. These include in the N-SEM (EC) group, *CBS* and DNA (cytosine-5-)-methyltransferase 3 beta (*DNMT3B*), and in the SEM group, *LBP-9*, *KIT*, *TCL1A*, *FRCP2* and *MMP9*. It is important, however, to keep in mind that Sperger *et al* identified genes specific for TGCT-derived cell lines compared to somatic cell lines and normal testis, whereas we compared expression in SEM and N-SEM against their precursor CIS cells. Therefore, our analysis did not identify some tumour markers such as *POU5F1*, *FLJ10884* and *AK3,* because they already were upregulated in the CIS cells. The difference in the experimental set-up thus probably largely explains the differences in the identified markers.

Interestingly, *DNMT3B* and DNA (cytosine-5-)-methyltransferase 3 like (*DNMT3L*), which are involved in the establishment of global DNA methylation in the mammalian genome (Burgers *et al*, 2002), were both found in the ‘Up in N-SEM’ group in the microarray analysis (Figure 1). The RT–PCR analysis verified the upregulation of *DNMT3B* in N-SEM samples, but not in the teratoma sample, suggesting that the signal originated from undifferentiated components such as EC (Figure 2). This is in line with previously described differences in the epigenetic phenotype of SEMs and N-SEMs. The genome of N-SEMs appears to be more methylated than that of both CIS and SEMs (Smiraglia *et al*, 2002; Smith-Sorensen *et al*, 2002; Honorio *et al*, 2003). Teratomas are more differentiated than EC and the absence of *DMNT3L* expression in teratomas in the RT–PCR analysis could indicate that a demethylation of DNA occurs when an EC differentiates into a teratoma.

The differential expression of DNA methyltransferases suggests that there may be epigenetic differences between the SEM and N-SEMs, which could play a central role in the progression of CIS cell into the different TGCT subtypes. If changes in methylation are implicated in the transformation into N-SEMs, demethylation could be highly tumour specific. On the cellular level, chromosomal region-specific demethylation may open for transcription of multiple transcripts from the same locus, which then could lead to the observed presence of multiple overlapping transcripts.

Combining the verified results of previous studies and the verified findings of our current investigation allows identification of a distinct gene expression ‘signature’ of classical SEM in comparison to N-SEM. Genes/antigens preferentially expressed in SEM include PLAP (Hofmann *et al*, 1989), M2A (Marks *et al*, 1995), KIT (Strohmeyer *et al*, 1991; Rajpert-De Meyts and Skakkebaek, 1994; this study), cyclin D2 (CCND2) (Houldsworth *et al*, 1997; Bartkova *et al*, 1999; Okada *et al*, 2003), MAGE-A4 (Aubry *et al*, 2001), HIWI (Qiao *et al*, 2002), DAZL-1 (Lifschitz-Mercer *et al*, 2002), *TFCP2L1* (this study), Aggrus (Kato *et al*, 2004) and JUP (Skotheim *et al*, 2003). The emerging expression pattern of N-SEMs is more complex, and because of histological variability of these tumours, a distinct ‘signature’ may not exist for this entity. The heterogeneity is especially prominent in teratomas, which express variable somatic cell-specific genes, and each component of the teratoma has to be studied separately. The undifferentiated EC and EC-derived cell lines have been more thoroughly investigated. A number of proteins highly expressed in EC are related to pluripotency, for example, POU5F1 (OCT-4) (Looijenga *et al*, 2003) and TFAP2C (Hoei-Hansen *et al*, 2004b), and are equally highly expressed in SEM. It is important to remember that the genes we have identified here are differentially expressed between both CIS, SEM and N-SEM. The list of genes/antigens expressed preferentially in EC *vs* SEM is thus rather short and only includes TRA-1–60 (Andrews *et al*, 1984), *KRT19* and *DNMT3L* (this study).

In conclusion, we have identified gene expression markers of the two major subtypes of TGCT and compared them to the gene expression profile of CIS using a genome-wide microarray. Identified markers were verified with both RT–PCR and ISH on a range of TGCT samples. Verification showed some interpatient variation, which is similar to that found in other studies, but when a range of genes was investigated, discrimination between the two tumour types was possible. The function of some of the identified genes confirmed that epigenetic differences exist between the major TGCT subtypes, the N-SEMs and SEMs, and that epigenetic factors most probably contribute to the phenotypic plasticity of TGCTs. It is, however, clear that we are only at the beginning of the road, and the understanding of the mechanism of neoplastic transformation and invasive progression of germ cell neoplasms requires further research.

## Figures and Tables

**Table 1:** Histological description and abbreviated names of the samples used

**Abbreviation**	**RT–PCR**	**Microarray**	**Histology**
50% CIS^a^		×	Approximately 50% tubules with CIS and 50% tubules with normal spermatogenesis. From the vicinity of a SEM
75% CIS^a^		×	Approximately 75% tubules with CIS and 25% tubules with normal spermatogenesis. From the vicinity of a SEM
100% CIS^a^		×	Tissue containing almost entirely CIS. From the vicinity of an embryonal carcinoma
SEM		×	Homogeneous classical SEM
EC, TER, YS (N-SEM)	×	×	N-sem consisting of embryonal carcinoma, teratocarcinoma and yolk sac tumour
SEM1	×		Classical SEM with some connective tissue
SEM2	×		Classical SEM with some atrophy
SEM3	×		Homogeneous classical SEM
TER1	×		Teratoma
EC1	×		Embryonal carcinoma, homogeneous
EC2	×		Embryonal carcinoma and CIS with some necrosis
CIS1 (SEM)	×		100% tubules with CIS (in the vicinity of a SEM)
CIS2 (EC)	×		70% tubules with CIS (in the vicinity of an embryonal carcinoma)
CIS3	×		90% tubules with CIS without an overt tumour, but microinvasion, perhaps progressing to a SEM
CIS4	×		90% tubules with CIS without an overt tumour, but microinvasion, perhaps progressing to a N-SEM
NOR1	×		Normal, complete spermatogenesis from a patient with prostate cancer
NOR2	×		Normal, complete spermatogenesis from the vicinity of an embryonal carcinoma

RT–PCR=reverse transcriptase–polymerase chain reaction; CIS=carcinoma *in situ*; SEM=seminoma; N-SEM=nonseminoma; EC=embryonic carcinoma.

a Expression values of CIS *vs* normal are from Almstrup *et al* (2004).

**Table 2:** Filtering of data to display genes specifically regulated in SEM and N-SEM compared to CIS

**Experiment**	**Up in SEM**	**Up in N-SEM**	**Down in SEM**	**Down in N-SEM**
N-SEM *vs* 100% CIS	<2	>5	>2	<0.5
SEM *vs* 100% CIS	>3	<2	<0.5	>2
SEM *vs* N-SEM	>2	<0.5	<0.5	>2
100% CIS *vs* Normal^a^	<2 and >0.5	<2 and >0.5	>2	>2

CIS=carcinoma *in situ*; SEM=seminoma; N-SEM=nonseminoma.

a From Almstrup *et al* (2004).
